# Oral non-Hodgkin’s lymphoma in a patient with rheumatoid arthritis treated with etanercept and methotrexate

**DOI:** 10.4317/jced.51922

**Published:** 2015-02-01

**Authors:** Eleni A. Georgakopoulou, Marina D. Achtari, Kostas Evangelou, Christos Kittas

**Affiliations:** 1 MD, DDS, MSc, PhD. Oral Medicine Specialist; 2 DDS, MS. Dental Clinic, General Hospital Paidon Pentelis, Athens, Greece; 3 MD, PhD, Pathologist. Department of Histology and Embryology, School of Medicine, University of Athens, Greece

## Abstract

Oral non-Hodgkin’s lymphomas (O-NHLs) are a rare group of diverse lymphoid tissue malignancies and represent less than 5% of the oral cavity malignancies and 2% of all extra-nodal NHLs. Oral-NHLs affect the Waldeyer’s-ring, the salivary glands, the bone of the jaws and the oral mucosa, their clinical appearance is very heterogeneous. Among the risk factors for NHLs are immunosuppression (primary or secondary), autoimmunity and inflammation. O-NHLs share the same risk factors. This case report describes a patient with O-NHL which was possibly linked to the combination of methotrexate and etanercept for the treatment of her rheumatoid arthritis. To our knowledge this is probably among the first cases of O-NHL with possible relation to the use of a Tumor Necrosis Factor (TNF) antagonist biological agent (etanercept). This case could contribute to the sensitization of the dentists for the signs and symptoms of this rare malignancy. It also underlines the need for thorough medical history and medication recording for all the dental patients.

** Key words:**Lymphoma (oral) methotrexate, etanercept.

## Introduction

Non-Hodgkin’s lymphomas [NHLs] are a heterogeneous group of lymphoid tissue malignancies accounting for at least 61 types of NHLs [WHO 2008 classification] (1). NHL has nearly doubled its incidence over the last 40 years becoming the fifth most common cancer (2,3). Lymphomas can occur outside the lymphatic system. The most common tissues of extra-lympatic/ extra-nodal origin are the mucosae [MALT lymphomas] especially of the gasrtointestinal tract and the skin [cutaneous lymphomas]. Extra-nodal involvement is identified in 30% of NHL cases (4). The NHL involvement in the oral cavity represents the 2% of cases that occur in an extra-nodal environment and is the 3rd most common oral cancer (5), but accounts for less than 5% of oral cancers, as approximately 90% of oral cancers are squamous cell carcinomas (6). NHLs in the oral cavity may affect sites where lymphoid tissue is present [tonsils, Waldeyer’s ring], the major salivary glands, the jaws and also the oral mucosa (6).

The possibility for NHL increases as a result of uncommon immune-disorders such as hereditary disorders of immune dysfunction, HIV/AIDS, and organ transplantation, while other well-known risk factors comprise, infectious agents, a familial trait and autoimmune disorders (7). Also there is a reported higher risk of lymphomas in patients treated with immune-modulating agents (7). Among these agents biologic agents blocking the func-tion of Tumor Necrosis Factor [TNF], include among their [U.S. Food and Drug Administration] FDA and [European Medicines Agency] EMEA registered side effects a risk for malignant lymphoma development, notably of uncommon NHL types (8). Etanercept is a fusion protein resembling TNF receptors type-II, which acts by blocking circulating TNF and lymphotoxin-a (8). It is approved for the treatment of Rheumatoid arthritis, Psoriatic arthritis, Ankylosing spondylitis, Moderate to severe plaque psoriasis and Severely active polyarticular juvenile idiopathic arthritis in children aged 2 years and older (8). Cases of lymphomas have been reported in adult patients with Rheumatoid arthritis and children with Juvenile idiopathic arthritis (9), and the FDA added a black box label for this reason, but still questions remain whether etanercept risk for NHL is higher than that attributed to the underlying disease used for (9).

## Case Report

**Figure 1:**
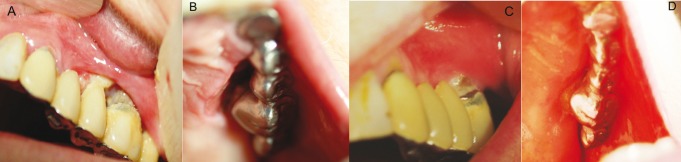
The initial appearance of the patient in May 2011 could represent an ulceration of various causes. Three years after diagnosis she is disease free. Note that she has inflammation on the buccal gingival area of #26 as she is reluctant to thoroughly brush this tooth. A) Buccal surface of Patient May 2011; B) Palatal surface of Patient May 2011; C) Buccal surface of Patient May 2014; D) Palatal surface of Patient May 2014.

**Figure 2:**
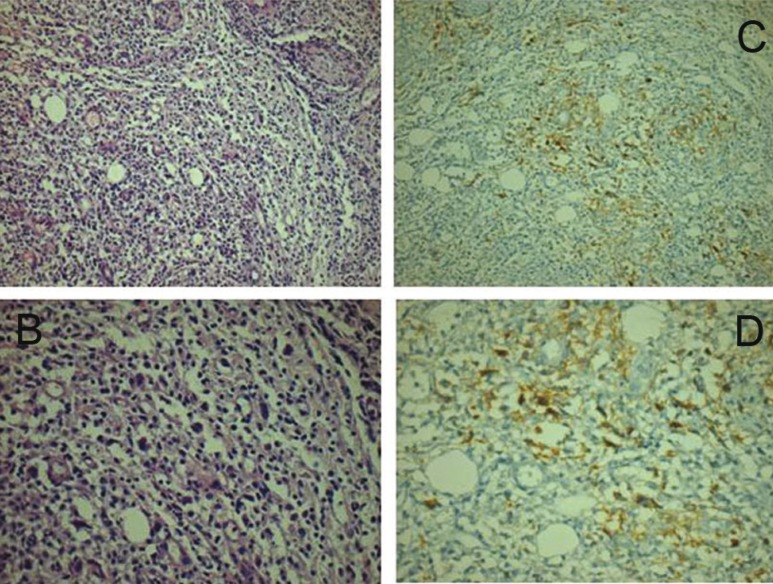
The histological (H/E) and the most preponderant immunohistochemical (IHC) findings showing lymphocytic infiltrates and CD30 positivity. A) Histology: H/E 200X; B) Histology: H/E 400X; C) IHC: H/E 200X; D) IHC: H/E 400X.

In this article we report a case of a 60 year old female patient with a ten year history of Rheumatoid arthritis treated with prednisolone 10mg/ daily, methotrexate 10mg /week, etanercept 25mg/weekly [for more than a year] and also risedronate sodium 35mg /weekly for prevention of osteoporosis. The main complaint of the patient was an ulcer with a 3 weeks reported duration, which caused moderate constant pain. She had done a full blood count and biochemical panel which showed no abnormal findings. She initially visited for this reason a general dentist who prescribed fluconazole and as the lesion did not resolve, she consulted an oral surgeon who suggested evaluation by an oral medicine specialist. The clinical examination revealed an ulcer with irregular margins covered with necrotic slough, located on the gingiva of the upper left quadrant [#25,#26], where the patient had an old bridge, (Fig. 1) and extended from the buccal to palatal surface. She had no palpable neck lymph nodes. According to the medical history and clinical findings the differential diagnosis, was : osteonecrosis, ulceration due to methotrexate, lymphoproliferative disorder of the oral soft tissue due to immune suppression. An immediate soft tissue biopsy [from the palatal surface] and orthopantomography were performed. The radiographic findings showed normal bone underneath the ulcer. The histological examination revealed lymphocytic infiltrate in hematoxylin eosin stain (Fig. 2), and a panel of immunohistochemistry markers for lymphoma revealed positivity for the CD30 [cluster of differentiation (Fig. 2) and Epstein–Barr virus encoded early RNA [EBER]. These findings were considered suggestive for B-cell origin NHL or lymphomatoid granulomatosis because of the presence of areas with angiocentric local allocation. Further molecular tests were scheduled [polymerase chain reaction [PCR]] and revealed monoclonal B-cell population among the lymphocytic infiltrate so, the final diagnosis was B-cell origin NHL.

The patient was referred to a tertiary oncology and hematology clinic [Laiko Hospital, Athens Greece] and was investigated with PET-scan [Positron emission tomography] and CT [Computed tomography], which showed that the only site affected was the oral mucosa. She was also evaluated by a Rheumatologist. Both the Hematologist and Rheumatologist in charge considered that her NHL was possibly related to the use of etanercept and secondary to methotrexate so they stopped her of the agents. The patient was treated with a combination of the anti-CD20 monoclonal antibody rituximab [Rituximab 1000mg / 6 months] and classic CHOP [Cytoxan, Hydroxyrubicin [Adriamycin], Oncovin [Vincristine], Prednisone],chemotherapy regimen. Three years after the initial diagnosis she is free of disease (Fig. 1), and her rheumatoid arthritis is controlled only with low dose prednisolone.

## Discussion

Despite the fact that etanercept has been labeled with a risk for NHL, the connection between the drug and the disease is still questionable (9). In our case the patient had an excellent response and we could not ignore the fact that her response may be related to the cessation of etanercept, a clinical observation reported in the literature (10). In addition, the fact that etanercept was combined with methotrexate, another agent linked to NHL risk [especially those related to Epstein–Barr virus] enhances the possibility that her NHL is rather related to her long term immunosuppressive therapy rather than her disease (7). To our knowledge this is among the first [if not the first] reported cases of O-NHL possibly related to etanercept or other anti-TNF agent (11). Patients with autoimmune diseases and long term immunosuppression should be informed to report their medical history to their dentist and the dentist should check their mouth for any unusual lumps or ulcers, as the clinical appearance of O-NHLs is highly atypical (6). The physical exam should be completed with palpation of neck nodes. Any abnormal findings should be carefully evaluated. The fact the patient’s dentist and oral surgeon did not attempt any extraction or periodontal surgery and suspected a non dental or periodontal cause for her ulcer contributed significantly to her excellent outcome (12).
